# A Few Words about the Plastic Materials

**Published:** 1881-05

**Authors:** Geo. A. Mills


### ARTICLE V.

### *A Few Words About the Plastic Materials.*

BY G. A. MILLS, BROOKLYN, N. Y.

The name of your paper indicates that you propose to give light; not that you intend to substitute the sunlight, or add more to it, but to throw in a few rays of helpfulness to those who do desire a luminous pathway in the practice of our specialty. How often the question is asked, vocally and mentally, what is the *best* material for filling teeth? As a principle, my answer would always be "*judgment.*" Materials of various kinds in the hands of skillful practitioners are made to serve a great purpose in arresting the disintegration of tooth tissue, and the standard of such (more often than is the common practice)

suggests the kind of materials. Some teeth in their tender age require very gentle methods of treatment, and the material that will expedite the operation and secure the best results under the circumstances, is the one to use. We all know the many difficulties attended with the practice of children from the ages of two to ten or thereabouts, yet they are very varied, and the time of presentation and circumstances will give us a fair field of what we have before us for this class of patients. Short and quick, compiled with as much comfort as possible, is the plan for securing the best results with young patients. My best judgment is that plastic materials will serve the best uses, yet no one of them are the *best* for all cases. None of them are all that we could desire. I do not give my patients the idea that because I have filled a tooth for a child that I may not have to renew the operation—perhaps it may require renewal often. I tell them I will do the best I can. Some ask, Is this or that filling temporary? I reply, It is, without any doubt; it may, however, remain, and serve the purpose required, for a longer or shorter period—possibly it will serve the full term of the tooth, be it a temporary one. The same prognosis applies, in a large degree, to all patients. Hill's stoppings are valuable when their use is indicated; oxychlorides the same. These have a quality that can be made to serve a useful purpose in a class of teeth that are deficient in calcification. The chloride of zinc acts as a source of irritation, and this serves to produce an increased vital action, and the result is the hardening of the dentine in contact with the filling. I am quite sure that many do not know by actual observation the fact I have stated, but abundance of proof can be furnished. My conviction is—gathered from actual practice—that a majority of young teeth, and even the teeth of more or less maturity, should have the value of this treatment, and we thus will be able to give more comfort and usefulness to teeth we labor to preserve; so this class of materials are still in demand, even more than ever, as their value comes to be recognized. The phosphate cements are in some respects an advance of the two named. They are more conveniently applied, and are generally more desirable. The putty-like quality is a decided advantage in applying in a large number of cases, being more readily controlled and its quicker setting properties, coupled with the greater resistance to friction, are additional qualities in its favor, and so far as it is wanting in the inotating effect, it is still another advantage. I have used nearly *all* of these preparations made in this country, commencing with Weston's, and with them all I have had varied results. So far I have found them *all* to be of help, and serve, here and there, some good purposes. Since Dr. J. H. Smith, o New Haven, Connecticut, has introduced his Phosphate Cement, which he has christened by the *hard* name of Adamantine, I have made quite an exclusive use of it, and have put it to some severe tests, by making *contour operations.* As a sample—one case: Two superior bicuspids on both proximal (largely) and grinding surfaces, and the molar joining one proximal and extensive grinding surface—all pulpless. I applied the dam, and after completing the operations I filled other teeth with gold, requiring about one hour's time; this gave advantage, so far as time is an advantage, in setting and keeping away the contact of moisture. In another mouth I made similar operations, applying the dam, and not using so much time. The first I made in the fall of 1870, or the winter, very soon after Dr. Smith introduced the material, (I have some of the first;) the second was in the summer of 1880. These operations remain unchanged—no perceptible wear. I have used this adamantine in a great variety of cases, and my experience with them gives me a decided preference for it.

My experience with oxychlorides has been in favor of mixing them into a creamy paste, and so be able, in the major part of the cases, to *squat* the material into the cavity and readily take the form of it, and it has seemed to secure a greater density of filling. With the Adamantine I have resorted to both the paste and the putty, and I am not able to discern a great amount of difference after I use the creamy form in the first part of the operation, and the putty in the latter, and getting *good* results when circumstances are considered.

The stickiness of the phosphates has been complained of, but occasionally dip the manipulating instrument into the dry powder, and there will be no difficulty. I will notice a *decided advantage* in the creamy paste when there is danger of too much pressure upon the pulp. As a foundation for gold fillings to be made in deep seated cavities, or for the prtaection from thermal shocks, the Adamantine is *truly* valuable, for it can be so readily introduced in the putty form and quickly sets.

For mixing, a porcelain platter and spatula are required. I put upon the platter, for large operations, or when time is to be required more than ordinarily, two portions of powder and liquid, so I may be able to change the proportions *ad libitum.* In simple cases I proceed accordingly, observing the general requirements for successful service. As time goes on and advance is made in the line of real knowledge, we may reasonably hope that we shall have *real* improvements in the list of plastic materials. There is, without a doubt, a larger range of usefulness in all the list of plastic materials than has yet been secured. According to the skill of the practitioner the wisdom in the choice of the several materials will be made manifest. To be wise should be the daily aspiration of every one who assumes the responsibility of serving those who apply to him.—*Dental Luminary.*

---

